# Randomized controlled trial of overall functional exercise process in perioperative of percutaneous transforaminal endoscopic discectomy

**DOI:** 10.1097/MD.0000000000032544

**Published:** 2022-12-30

**Authors:** Shuang Wang, Hai-Long Yu, Liang Zheng, Jun-Xiong Ma, Hong Wang, Liang-Bi Xiang, Yu Chen

**Affiliations:** a Department of Orthopedics, General Hospital of Northern Theater Command, Shenhe District, Shenyang, China.

**Keywords:** endoscope, exercise, lumbar disc herniation (LDH), minimally invasive treatment, transforaminal

## Abstract

Percutaneous transforaminal endoscopic discectomy (PTED) has become the standard surgery for the patients of lumbar disc herniation with the advantages of less trauma and rapid recovery. But still some patients have poor prognosis after PTED. A major risk factor associated with the poor prognosis may be the unfit function exercise.

**Objective:**

To discuss the effect of overall functional exercise process for PTED.

**Methods:**

In January 2019 to June 2020, a single center randomized controlled trial was proceeded. The patients scheduled for PTED were randomly divided into the experimental group, which received overall functional exercise and the control group, which received routine process. The overall process included advance, whole-course exercise and integrating of traditional Chinese medical methods. The general information, visual analog scale (VAS) score and Oswestry Dysfunction Index (ODI) score at each follow-up point perioperative period were compared between the 2 groups.

**Results:**

There were no significant differences in the general information, the preoperative VAS and ODI. On the 3rd day after operation, the VAS of low back pain and leg pain in the experimental group were lower than the control group. One month after operation, the VAS of low back pain in the experimental group was lower than that in the control group. One to 3 months after operation, the ODI scores of the experimental group were better than that of the control group. There was no significant difference in modified MacNab index between the experimental group and the control group.

**Conclusion:**

Function exercise is important for the prognosis of minimally invasive lumbar surgery. The overall function exercise process perioperative is helpful to relieve the short-term pain of the patients and significantly improve the prognosis.

## 1. Introduction

Lumbar disc herniation (LDH) is one of the most common diseases of the department of orthopedics, which could cause the pain of the low back and the legs. Also, the symptoms include sensorimotor dysfunction of the lower limbs. For most patients, the conservative treatments such as restricting activity, non-steroid anti-inflammatory drug and physical therapy could receive ideal therapeutic effect. Meanwhile, the patients with poor effect should be treated with operations. With the progress of the surgery and technology, minimally invasive operations have been applied in the treatment of LDH. Among all the minimally invasive techniques, percutaneous transforaminal endoscopic discectomy (PTED) has gradually become the most practical and effective method with the advantages of less trauma and rapid recovery, which is performed through a narrow working channel and preventing the breaking to the surrounding stable structures. The practice of these years has shown that the operation of PTED has the advantages of limited trauma and blood during the operation, shorter hospital stays and less hospitalization costs.

Although with the advantages above, there are still some patients with poorer prognosis. These poor prognoses include residual lower limb symptoms such as pain or numb, unsatisfactory relief of the low back pain.^[1]^ With these poor prognoses, the patients might be miserable about the operation and the surgeon. The reasons about the poor prognoses may be associated with several aspects, among which, the function exercise is an important but often neglected one. Related studies have shown that these poor prognoses may be associated with improper or inadequate function exercises. Also, the reasons associated with the poorer condition of function exercise might be multiple, for example the fear of pain, or being unfamiliar with the function exercise. Some researchers have approved that the introductions and educations of the function exercise are important for the implementation of the exercise. Only with the detailed education and the thorough understanding of the exercises, the patients could grasp the process of the function exercise enough and apply it effectively. For this reason, we formulated a whole functional exercise process and carried on through the perioperative period of PTED. This process included advanced exercise, whole-course exercise, integrated traditional Chinese medicine and individual exercise.

## 2. Methods

### 2.1. Trial design: single center randomized controlled trial

In January 2019 to June 2020, the research was proceeded in the Department of Orthopedics, General Hospital of Northern Theater Command. The patients with the diagnoses of LDH and meeting the indications of PTED were chosen for the current research. Then the patients were screened by the inclusion and exclusion criteria. The inclusion criteria: the symptoms were low back pain with unilateral lower limb radiation pain, with or without numbness; computed tomography, magnetic resonance imaging, and other imaging examination showed single segmental LDH; symptoms, physical examination results were consistent with the imaging results; underwent the regular conservative treatment for at least 1 month and the symptoms were not relieved or aggravated; and complete at least 12 months of follow-up. The exclusion criteria: symptoms, physical examination results are not consistent with imaging results; multi-segmental herniated intervertebral disc; complicated with lumbar deformity, spinal stenosis, spondylolisthesis, instability, herniated disc calcification; and previous surgical history of the same site. After the screening, the subjects were selected and simple randomization divided into the experimental group and control group using the random number method. The corresponding author generated the random allocation sequence. All the subjects underwent PTED performed by the same surgeon. The control group was given routine exercise during the perioperative period, while the experimental group was given integrated functional exercise flow. The current research has received ethics approval from the committee of the General Hospital of Northern Theater Command. The corresponding author generated the random allocation sequence, the first authors enrolled participants. Then the first authors and one of another author WH performed the research.

### 2.2. Interventions

#### 1.2.2.. The process of function exercise.

**Figure 1.**
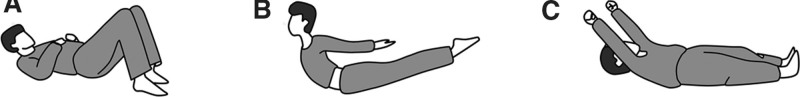
Diagrammatic drawing of functional exercise. (A) The movement of five-point supporting. (B) The movement of swallow-flying. (C) The movement evolving from the traditional Chinese medicine exercises “Ba-duan-jin”.

For the experimental group, a function exercise guiding group was assigned to manage the functional exercises, which included the surgeon and nurse in charge of the patient. Meanwhile, the functional exercise began at the time of admission and lasted until discharge. As for the exercise actions, on the base of the routine actions such as “five points supporting” (Fig. 1A) and “flying as swallow” (Fig. 1B), an action (Fig. 1C) was added. This action actually was evolved from the traditional Chinese medicine exercises “Ba-duan-jin.” The exact action included the following steps, slowly raising the straight upper limbs overhead and straightening the lower limbs, extending the hands and feet to the cephalad side and caudal side, respectively, as far as possible, maintaining about 5 seconds, then relaxing and taking back the limbs on the bed to rest 5 seconds. All these steps were 1 cycle, every exercise cycle included 5 to 8 cycles according to the condition of the patient. The frequency of daily exercises and the times of the movements for every exercise were determined by the surgeon and the nurse according to the exact condition of the patient.

For the control group, routine exercise program was applied including the movements of “five-point supporting” (Fig. 1B) and “swallow-flying” (Fig. 1C), and the exercise began the next day after operation. Also being different from the experimental group, the exercises began from the first day after the operation as routine.

#### 2.2.2.. Operation method.

All operations were performed under local anesthesia by the same surgeon. The patients were made to remain in prone position, with the joints of hips and knees slightly bent. C-arm fluoroscopy was used to locate the target intervertebral space and determine the puncturing point and direction. After the dilatation cannula and working cannula were placed successively, the foraminal endoscope was connected and discectomy was performed.

### 2.3. Outcomes

#### 1.2.3.. Evaluation of the prognosis.

Referring to the related literature, the sample size was determined. Also, the visual analog scale (VAS) was chosen to evaluate the pain of waist and lower limbs. The lumbar function was evaluated by Oswestry Dysfunction Index (ODI) score. The time points of evaluation were pre-operation, 3rd day after the operation (VAS), and 1, 3, 6, and 12 months after the operation. At each postoperative follow-up point, whether including the items associated with travel was determined according to the patients’ condition. At the last follow-up time point, the therapeutic effect was evaluated by the improved MacNab standard.

### 2.4. Statistical analysis

The data were collected and analyzed using GraphPad Prism 8 (GraphPad Software, https://www.graphpad-prism.cn/). The measurement data were expressed as mean ± standard deviation, and the counting data were expressed as percentage or numerical value. Independent sample *t* test was used to compare the measurement data of general data and the chi-square test was used to compare the count data of general data. Paired *t* test was applied to compare the scores of VAS, ODI and. Rank sum test was used when the data were non-normal distribution or uneven variance. The difference was statistically significant when *P* < .05. Data analyses were performed by another assistant not associated with the current research.

## 3. Result

### 3.1. Participant flow

Totally, 172 patients were eligible in the period and 25 were excluded for the reason of not meeting the inclusion criteria. Then the remaining 147 patients allocated were divided into the 2 groups. In the period of follow-up, 8 patients in the experimental group and 10 patients in the control group were lost. Ultimately, the experimental group included 63 cases patients, including 34 males and 29 females, with an average age of 38.7 ± 7.6 (19–64), the average course of disease from onset to operation was 14.7 ± 5.9 (4–47) months, the distribution of protruding segments were 7 cases of L3-4, 41 cases of L4 to 5, 15 cases of L5 to S1, and the average period of follow-up was 17.2 ± 4.2 (14–31) months. The control group included 66 cases, including 38 males and 28 females, with an average age of 35.2 ± 8.1 (20–67) years, the course of disease from onset to operation was 15.2 ± 4.9 (2–43) months, the distribution of protruding segments were 6 cases of L3 to 4, 44 cases of L4 to 5, 16 cases of L5 to S1, and the average period of follow-up 18.8 ± 4.1 (14–33) months. There were no differences between the 2 groups in the general data.

### 3.2. Evaluation of the prognosis

Before the operation, there were no significant differences between the 2 groups in the VAS score, ODI scores of low back or leg pain. Then, on the 3rd day after the operation, the VAS score of low back pain and leg pain in the experimental group was significantly lower than the control group. Also, on the follow-up point of 1 month after operation, the VAS score of low back pain in the experimental group was lower than the control group. There were no significant differences in VAS of low back pain or leg pain between the 2 groups at 3, 6 and 12 months after operation (*P* > .05). (Table 1).

**Table 1:** Comparison of VAS.

Follow-up time point	Low back pain		Leg pain	
	Experimental group	Control group	Experimental group	Control group
Pre operation	3.2 ± 1.3	3.5 ± 1.4	6.7 ± 2.5	7.2 ± 1.9
3rd d post operation	2.1 ± 1.1*	2.6 ± 0.9*	2.3 ± 1.2***	3.1 ± 0.9***
1 mo post operation	1.8 ± 0.7**	2.1 ± 0.8**	1.9 ± 1.0	2.2 ± 1.3
3 mo post operation	1.5 ± 1.0	1.6 ± 0.9	1.5 ± 0.9	1.6 ± 1.0
6 mo post operation	1.5 ± 0.9	1.5 ± 1.1	0.8 ± 0.5	0.9 ± 0.4
12 mo post operation	1.4 ± 1.1	1.3 ± 0.8	0.8 ± 0.4	0.9 ± 0.3

VAS = visual analog scale.

* ,

** , and

*** mean that *P* < .05.

For the ODI scores, the results were better in the experimental group than the control group at 1 and 3 months after operation, then there were no significant differences at 6 and 12 months (Table 2).

**Table 2:** Comparison of ODI.

Follow-up time point	ODI	
	Experimental group	Control group
Pre operation	71.3 ± 14.2	68.9 ± 15.4
1 mo post operation	23.7 ± 5.6*	27.8 ± 6.8*
3 mo post operation	17.5 ± 6.9**	21.6 ± 7.1**
6 mo post operation	11.2 ± 2.1	10.9 ± 1.1
12 mo post operation	9.2 ± 1.4	9.3 ± 0.8

ODI = Oswestry Dability Index.

* , and

** mean that *P* < .05.

The excellent and good rate of the last follow-up was evaluated by the modified MacNab index, and there was no significant difference between the experimental group (92.1%) and the control group (90.1%).

## 4. Discussion

In the current research, the participants of the experimental group exhibit preferred prognoses than the control group. These prefer prognoses mean less pain on the 3 day and 1 month after the operations. Meanwhile, the value of ODI was better at 1 and 3 months after the operation. It seems that the overall functional exercise process could obviously improve the prognoses of PTED. Since Kambin^[2]^ used endoscopic surgery to treat LDH in the 1990s, the related techniques have been improved continuously. Yeung^[3]^ and Hoogland^[4]^ respectively developed different puncturing techniques as Yeung Endoscopic Spine System and Transforaminal Endoscopic Spine System. The core of these 2 techniques is to establish working channels through the Kambin triangle^[5,6]^ through the intervertebral foramen. Then, the nerve roots were relieved fully from the compression of other structures such as the herniated intervertebral disc. These kinds of surgical techniques are usually referred as PTED. After the development of nearly 30 years, PTED surgery has been widely used in the treatment of LDH^[7,8]^ and achieved desired results.^[9–11]^ Meanwhile, the theory, surgical technology, and equipment related to PTED have been fully developed to ensure the prognosis of the operation.

Although achieving the above achievements, there are also some patients with poor prognosis after PTED, which were related with persistent low back pain or poor relief of lower limb. The causes of these poor prognoses may originate from many aspects,^[12]^ among which the lack of adequate and appropriate functional exercise might be an important reason. Some studies have shown that the soft tissues around the lumbar vertebrae are of importance to the occurrence and development of the low back pain.^[12]^ Then, adequate and appropriate functional exercise could relieve the pain of the soft tissue and create ideal conditions for the recovery postoperatively. Formerly, functional exercises often related to conservative treatments of the disease or injuries of spine. In recent years, increasing number of studies have shown that proper rehabilitation and exercise postoperative of the lumbar surgeries can relieve the pain^[13]^ and promote the courses of recovery.^[14–16]^ Especially for the patients undergoing minimally invasive lumbar surgery, appropriate functional exercise can promote the advantages of minimally invasive surgery and improve the prognosis of the patients.^[17,18]^ Meanwhile, some new understandings of the function exercises were raised. For example, some surgeons considered that not only the postoperative functional exercise was important to the prognosis, but the preoperative function exercise combined with health education^[19]^ was also helpful to improve the prognosis. Therefore, the exact flaw of the function exercise has become important issues such as the kind and time of every movement included in the course of exercise.

For the consideration above and combining the concept of enhanced recovery after surgery, the current research formulated the perioperative overall functional exercise process for patients with PTED. The process emphasized “advanced exercise,” “whole-course exercise,” ’individual exercise’ and integrated traditional Chinese medicine. Advanced exercise means that the patients begin the function exercise pre-operatively. The exercise was performed under the guidance of physicians and nurses in charge, beginning as soon as the admission in order to form the muscle memory. Also, the exercises beginning pre-operatively could make the patients familiar with the feeling of related muscle and the body reaction after different times of repetition of each exercise action. Full-course exercise means the function exercise that is conducted throughout the perioperative period. The exercise was carried out as soon as possible after the operation, which began from subtle to obvious, from isometric contraction to flexion and extension of the joints, from less to more, and from simple to complex. The integration of traditional Chinese medicine means that some movements were evolved from the traditional Chinese medicine into the exercise process. Individualized exercise refers to the function exercise guiding group composed of physicians and nurses in charge formulating the exercise mode of each patient throughout the process, and flexibly adjusting the exercise program according to the physique, symptom severity, and reaction after exercise. Meanwhile, the process of function exercise after discharge should be performed from the course of the exercise in the hospital. When the patients were followed up regularly, the functional exercise guidance group adjusted the exercise plan according to the recovery.

The limitations of the current research are as follows: the number of samples included is limited, so the conclusions of the research need to be tested by a larger sample control experiment; the evaluation index of curative effect is limited; and the process of overall functional exercise needs to be further improved, such as adding more movements and methods that can promote recovery, further refining the frequency and number of function exercise.

As the conclusion, we considered that the overall functional exercise in the perioperative period of PTED plays a positive role in relieving short-term pain and improving the prognosis of patients. Also, this process of exercise could improve the advantages of minimally invasive spinal surgery such as PTED.

## Author contributions

**Conceptualization:** Yu Chen.

**Data curation:** Shuang Wang, Liang Zheng, Jun-Xiong Ma, Hong Wang.

**Formal analysis:** Liang Zheng.

**Investigation:** Shuang Wang, Jun-Xiong Ma.

**Supervision:** Liang-Bi Xiang, Yu Chen.

**Writing – original draft:** Shuang Wang, Jun-Xiong Ma.

**Writing – review & editing:** Hai-Long Yu, Liang-Bi Xiang, Yu Chen.
